# Radiotherapy related skin toxicity (RAREST-01): Mepitel® film versus standard care in patients with locally advanced head-and-neck cancer

**DOI:** 10.1186/s12885-018-4119-x

**Published:** 2018-02-17

**Authors:** Carlos Narvaez, Claudia Doemer, Christian Idel, Cornelia Setter, Denise Olbrich, Zaza Ujmajuridze, Jesper Hansen Carl, Dirk Rades

**Affiliations:** 1 Department of Radiation Oncology, University of Lübeck, Ratzeburger Allee 160, D-23562 Lübeck, Germany; 2 Departments of Radiation Oncology and Oto-Rhino-Laryngology and Head and Neck Surgery, University of Lübeck, Lübeck, Germany; 3 Department of Radiation Oncology, Christian-Albrechts University Kiel, Kiel, Germany; 4 Centre for Clinical Trials Lübeck, Lübeck, Germany; 5 Department of Oncology, Zealand University Hospital, Naestved, Denmark

**Keywords:** Head-and-neck cancer, Radio(chemo)therapy, Radiation dermatitis, Mepitel® film, Standard care

## Abstract

**Background:**

The aim of the present trial is to investigate a new option of skin protection in order to reduce the rate of grade ≥ 2 skin toxicity in patients receiving radiotherapy alone or radiochemotherapy for locally advanced squamous cell carcinoma of the head-and-neck (SCCHN).

**Methods / Design:**

This is a randomized, active-controlled, parallel-group multi-center trial that compares the following treatments of radiation dermatitis in patients with head-and-neck cancer: Mepitel® Film (Arm A) vs. standard care (Arm B). The primary aim of this trial is to investigate the rate of patients experiencing grade ≥ 2 radiation dermatitis (according to Common Toxicity Criteria for Adverse Events (CTCAE) Version 4.03) until 50 Gy of radiotherapy. Evaluation until 50 Gy of radiotherapy has been selected as the primary endpoint, since up to 50 Gy, the irradiated volume includes the primary tumor and the bilateral cervical and supraclavicular lymph nodes, and, therefore, is similar in all patients. After 50 Gy, irradiated volumes are very individual, depending on location and size of the primary tumor, involvement of lymph nodes, and the treatment approach (definitive vs. adjuvant). In addition, the following endpoints will be evaluated: Time to grade 2 radiation dermatitis until 50 Gy of radiotherapy, rate of patients experiencing grade ≥ 2 radiation dermatitis during radio(chemo)therapy, rate of patients experiencing grade ≥ 3 skin toxicity during radio(chemo)therapy, adverse events, quality of life, and dermatitis-related pain. Administration of Mepitel® Film will be considered to be clinically relevant, if the rate of grade ≥ 2 radiation dermatitis can be reduced from 85% to 65%.

**Discussion:**

If administration of Mepitel® Film instead of standard care will be able to significantly reduce the rate of grade ≥ 2 radiation dermatitis, it could become the new standard of skin care in patients irradiated for SCCHN.

**Trial registration:**

clinicaltrials.gov NCT03047174. Registered on 26th of January, 2017. First patient included on 9th of May, 2017.

## Background

Locally advanced squamous head-and-neck cancer is a serious malignant disease. In about 90% of head-and-neck cancers, the histology is squamous cell carcinoma (SCCHN). The vast majority of patients with locally advanced SCCHN receive radiotherapy, either as a part of a definitive treatment approach, or as an adjuvant treatment following surgery. If radiotherapy is administered as definitive treatment, it is usually combined with concurrent cisplatin-based chemotherapy [1]. In an adjuvant situation, chemotherapy will be added to radiotherapy in case of risk factors, namely microscopically or macroscopically incomplete resection or in case of extra-capsular spread of lymph nodes metastases.

Radiotherapy of locally advanced SCCHN may be associated with severe acute toxicities including skin reaction such as erythema or desquamation. Skin toxicity is enhanced if concurrent chemotherapy is administered. If the skin toxicity becomes severe (grade ≥ 3 according to the Common Toxicity Criteria for Adverse Events (CTCAE) Version 4.03), it may lead to a reduction of the planned chemotherapy dose and to interruptions of radiotherapy. Interruptions of radiotherapy have been reported to be associated with poorer treatment outcomes in patients with SCCHN [2, 3].

In order to successfully avoid grade ≥ 3 skin toxicity, it appears mandatory to avoid or at least postpone the development of grade 2 skin toxicity. In previous studies of patients receiving radiotherapy or radio(chemo)therapy for locally advanced head-and-neck cancer, rates of grade ≥ 2 skin toxicity ranging between 86% and 92% have been reported, although the standard procedures of skin care and protection had been applied [1, 4, 5]. These figures demonstrate that the results of standard care need to be improved.

A few years ago, the results of a systematic inpatient controlled clinical trial were published that had investigated the use of an absorbent, self-adhesive dressing (Mepilex® Lite) for skin protection in patients irradiated for breast cancer [6]. According to this study the dressings were able to significantly reduce the radiation-related skin erythema. Similar dressings (Mepilex® Border Sacrum and Mepilex® Heel dressings) have been demonstrated in a randomized trial to be effective also in the prevention of sacral and heel pressure ulcers in trauma and critically ill patients [7]. In another randomized trial a silver-containing soft silicone foam dressing (Mepilex® Ag dressing) was as effective in the treatment of partial-thickness thermal burns when compared to the standard care (silver sulfadiazine) [8]. In addition, the group of patients treated with the Mepilex® Ag dressing demonstrated decreased pain and lower costs associated with treatment. More recently, a new dressing (Mepitel® Film) has been developed, which is thinner, softer and more comfortable than previous dressings.

The rationale for the present study is to investigate a new option of skin protection in order to reduce the rate of grade ≥ 2 skin toxicity in patients receiving radiotherapy alone or radiochemotherapy for locally advanced SCCHN.

## Methods / Design

### Endpoints of the study

The primary aim of this randomized multi-center trial is to investigate the rate of patients experiencing grade ≥ 2 radiation dermatitis (CTCAE v4.03) until 50 Gy of radiotherapy. Evaluation until 50 Gy of radiotherapy has been selected as the primary endpoint, since up to 50 Gy, the irradiated volume includes the primary tumor and the bilateral cervical and supraclavicular lymph nodes, and, therefore, is similar in all patients. After 50 Gy, irradiated volumes are very individual, depending on location and size of the primary tumor, involvement of lymph nodes, and the treatment approach (definitive vs. adjuvant).

In addition, the following endpoints will be evaluated:

1. Time to grade 2 radiation dermatitis until 50 Gy of radiotherapy
2. Rate of patients experiencing grade ≥ 2 radiation dermatitis during radio(chemo)therapy
3. Rate of patients experiencing grade ≥ 3 skin toxicity during radio(chemo)therapy
4. Adverse Events
5. Quality of life: Evaluation prior to radiotherapy, at the end of radiotherapy weeks 3 + 5, and at 3 weeks following radiotherapy
6. Pain: Evaluation prior to radiotherapy, at the end of radiotherapy weeks 3 + 5, and at 1 and 3 weeks following radiotherapy

### Study design

This is a randomized, active-controlled, parallel-group trial, which will compare the following treatments of radiation related skin toxicity in patients with head-and-neck cancer:

Mepitel® Film (Arm A) vs. Standard Care (Arm B).

The recruitment of all 168 patients should be completed within 24 months. The follow-up period will be 3 weeks. Stratification will be done using the following factors:

1. Tumor site: oropharynx/oral cavity vs. hypopharynx/larynx
2. Treatment approach: radiochemotherapy vs. radiotherapy alone
3. Participating site

### Inclusion criteria

1. Histologically proven locally advanced squamous cell carcinoma of the head-and-neck (SCCHN)
2. Conventionally fractionated (5 × 2 Gy per week) definitive or adjuvant radio(chemo)therapy
3. Age ≥ 18 years
4. Written informed consent
5. Capacity of the patient to contract

### Exclusion criteria

1. N3 stage (lymph nodes > 6 cm)
2. Distant metastases (M1)
3. Pregnancy, Lactation
4. Treatment with EGFR-antibodies (either given or planned)
5. Expected non-compliance

### Treatment

#### Radiotherapy

Radiotherapy is administered using conventional fractionation (5 × 2.0 Gy per week). The initial target volume includes the region of the primary tumor plus bilateral cervical and supraclavicular lymph nodes up to 50 Gy. Patients treated with adjuvant radiotherapy following complete resection of the primary tumor and the involved lymph nodes receive a radiation boost of 10 Gy (5 × 2.0 Gy per week) to the regions of the primary tumor and the involved lymph nodes. In case of a microscopically incomplete resection, the boost dose to the primary tumor region is 16 Gy. In case of extra-capsular spread (ECS) of lymph nodes, the lymph nodes showing ECS receive an additional boost of 6 Gy (i.e. a cumulative boost dose of 16 Gy). Patients receiving definitive radiotherapy, receive a boost of 10 Gy (5 × 2.0 Gy per week) to the primary tumor, the involved lymph nodes, and the lymph node levels adjacent to the involved lymph nodes. An additional boost of another 10 Gy (5 × 2.0 Gy per week) is administered to the primary tumor and the involved lymph nodes. Treatment should be performed as intensity-modulated radiotherapy (IMRT) or volumetric modulated arc therapy (VMAT) radiotherapy.

#### Chemotherapy

In patients who receive definitive radiotherapy, concomitant chemotherapy with cisplatin is administered. The cumulative cisplatin dose at the end of the fifth week of radiotherapy (50 Gy) should be 200 mg/m2. This cumulative dose may either be achieved with 20 mg/m2 given with radiotherapy fractions 1–5 and 21–25, 25 mg/m2 given with radiotherapy fractions 1–4 and 21–24, or weekly doses of 40/m2.

#### Skin care

##### Arm a (Mepitel® film)

Starting on the first day of radiotherapy, Mepitel® Film will be applied. Skin care will be continued until the end of the study period or until a patient experiences grade ≥ 2 moist desquamation or grade ≥ 3 radiation dermatitis. In case of grade ≥ 2 moist desquamation or grade ≥ 3 radiation dermatitis, antiseptic agents will be used daily followed by administration of silicon or calcium alginate bandage until moist desquamation disappears and radiaton dermatitis improves to grade 2.

##### Arm B (standard care)

Standard skin care will be applied starting on radiotherapy day 1 including fatty cream with 2–5% urea (fatty cream alone, if patients do not tolerate urea) and mometasone furoate cream. The treatment will be continued until the end of the study period or until a patient experiences grade ≥ 2 moist desquamation or grade ≥ 3 radiation dermatitis. In case of grade ≥ 2 moist desquamation or grade ≥ 3 radiation dermatitis, the same skin care regimen is used as in Arm A.

### Assessments

The following parameters will be recorded at the start of the trial: Medical history, physical examination, complications from head-and-neck surgery, age, gender, performance status, site of primary tumor, tumor stage, histology, HPV-status, histologic grading, surgery of primary tumor, extent of resection, neck dissection, complications of surgery, chemotherapy planned, skin status of the head-and neck region, and quality of life.

The following parameters will be assessed continuously throughout the course of the trial:

1. Radiation dermatitis: Radiation dermatitis will be assessed by two independent observers (specially trained nurses, technicians, or physicians) prior to radio(chemo)therapy, daily during radio(chemo)therapy and up to three weeks following radio(chemotherapy) according to CTCAE v4.03. If the graduation of radiation dermatitis varies between the two observers, skin toxicity will be assessed by an additional observer. Observers are required to be very experienced in rating skin reactions and will additionally undergo a particular briefing prior to the start of this study.
2. Adverse Events: Adverse events, other than radiation dermatitis will be assessed on an ongoing basis according to CTCAE v4.03.
3. Quality of life: Quality of life will be assessed prior to radio(chemo)therapy, at the end of radiotherapy weeks 3 and 5, and at three weeks following radio(chemo)therapy using EORTC QLQ-C30 Version 3.0 and EORTC QLQ-H&N35.
4. Pain: Dermatitis-related pain is assessed with a visual analogue scale (self-assessment: from 0 = No pain; 1 = Mild pain to 10 = Very severe pain) prior to, daily during and up to three weeks following radio(chemotherapy). Pain scores will be correlated with grade of skin reactions.

### Sample size calculation

The primary goal of this randomized trial is to demonstrate that Mepitel® Film is superior to Standard Care with respect to prevent grade 2 radiation dermatitis in patients receiving radio(chemo)therapy up to 50 Gy for locally advanced SCCHN. The null hypothesis of equal rates of grade ≥ 2 skin toxicity is tested against the two-sided alternative hypothesis of different rates. Based on this hypothesis system, the sample size required for this trial is calculated taking into account the following assumptions:

- A Chi-square Test will be applied
- The two-sided significance level is set to 5%
- In patients treated with radio(chemo)therapy for locally advanced SCCHN, previous studies have suggested rates of grade ≥ 2 skin toxicity of 86–92% if standard skin care was administered.
- Based on these data, a rate of grade ≥ 2 skin toxicity of 85% can be assumed in the reference group (“worst-case”), i.e. in patients receiving standard care for skin toxicity.
- Administration of Mepitel® Film will be considered to be clinically relevant, if the rate of grade ≥ 2 skin toxicity can be reduced to 65%.
- The power to yield statistical significance if the difference in rates is in fact 20 percentage points is set to 80%.

Based on these assumptions, 80 patients are required per study arm within the full analysis set. Taking into account that 5% of patients will not qualify for the set, a total of 168 patients should be randomized.

The rates of patients experiencing grade ≥ 2 radiation dermatitis in patients receiving radio(chemo)therapy up to 50 Gy will be statistically compared using the Cochran-Mantel-Haenszel Chi-square test on a two-sided significance level of 5%. This test is the natural non-parametric extension of the Chi-square test for testing the treatment effect, while adjusting for the effects of the stratification variables used for randomization. For further assessment of the robustness of the results, a logistic regression model for grade ≥ 2 radiation dermatitis will be applied including the parameters used for stratification. In addition, a model including also additional patient characteristics will be fitted. The confirmatory evaluation will be performed within the Full Analysis Set, the Per Protocol Set serves for further sensitivity analyses.

## Discussion

Radiotherapy is the most frequently administered treatment modality in patients with locally advanced SCCHN. A considerable number of these patients receive concurrent chemotherapy, generally including cisplatin or carboplatin [1]. Many of these patients, particularly those receiving radio-chemotherapy, experience severe acute side effects including radiation dermatitis. Severe skin reactions may require an interruption of the radiotherapy series, which can lead to a worsening of the patients’ prognoses. On multivariate analyses of a retrospective study of 153 patients irradiated for SCCHN, better overall survival was significantly associated with no interruptions of radiotherapy longer than one week (relative risk: 2.59, 95% confidence interval: 1.15–5.78, *p* = 0.021) [2]. So was local control (relative risk: 3.32, 95% confidence interval: 1.26–8.79, *p* = 0.015). In a SEER database analysis, patients irradiated for larynx cancer with an interruption of their radiotherapy had a 68% (95% confidence interval: 41% to 200%) increased risk of death than those patients without an interruption. Patients with head-and-neck cancers at other sites showed similar associations. However, due to the relatively small numbers of patients, the difference between patients who did and who did experience interruptions of radiotherapy did not reach significance [3]. To avoid such interruptions of radiotherapy due to radiation dermatitis, it is reasonable to avoid or at least significantly postpone grade 2 skin reactions. This appears a challenge for radiation oncologists, since in previous studies grade ≥ 2 radiation dermatitis occurred in 86% to 92% of patients, despite administration of standard skin care procedures from the first day of radiotherapy [1, 4, 5]. Therefore, skin care in patients irradiated for SCCHN needs to be improved, particularly to avoid interruptions of radiotherapy and a subsequent impairment of the patient’s prognoses in terms of local control and overall survival [2, 3]. The use of an absorbent, self-adhesive dressing represents a promising approach. According to a systematic inpatient controlled clinical, such dressings can significantly decrease radiation-related erythema of the skin in breast cancer patients [6]. Promising results have also been reported for the prevention of sacral and heel pressure ulcers in trauma and critically ill patients and the treatment of partial-thickness thermal burns [7, 8]. More recently, a new dressing named Mepitel® Film was developed that is thinner and softer than previous dressings and, therefore, appears more comfortable for the patients than the previous dressings. The randomized RAREST-01 compares this new dressing to standard procedures of skin care in patients with locally advanced SCCHN receiving radiotherapy or radiochemotherapy. If Mepitel® Film can significantly reduce the rate of grade ≥ 2 radiation dermatitis in patients irradiated for locally advanced SCCHN it would have the potential to become the new standard of skin care in this group of patients.

## Abbreviations

CTCAE: Common Terminology Criteria for Adverse Events
ECOG: Eastern Cooperative Oncology Group
ECS: Extra-capsular spread
EORTC: European Organisation for Research and Treatment of Cancer
IMRT: Intensity-modulated radiotherapy
QLQ: Quality of life questionnaire
SCCHN: Squamous cell carcinoma
VMAT: Volumetric modulated arc therapy
